# HIV Concealment and Victimization Among Adolescents with HIV (AWH): The Moderating Role of Emotional Social Support

**DOI:** 10.1007/s10461-026-05138-x

**Published:** 2026-04-24

**Authors:** Amrita Gill, Aditi Bhome, David Seal, Patricia Kissinger, Rohidas Borse, Manoj Pardeshi, Nishi Suryavanshi, Katherine P. Theall

**Affiliations:** 1 Department of Psychiatry and Human Behavior, Warren Alpert Medical School, Brown University, Box G-BH, Providence, RI 02912 USA; 2 KEM research hospital, Pune, India; 3 Department of Social, Behavioral, and Population Sciences, Celia Scott Weatherhead School of Public Health and Tropical Medicine, New Orleans, LA USA; 4 The Network of Maharashtra People with HIV, Pune, India; 5 Byramjee Jeejeebhoy Government Medical college, Pune, India; 6 Byramjee Jeejeebhoy Government Medical College - Johns Hopkins University Clinical Trials Unit, Pune, India; 7 Department of Epidemiology, Celia Scott Weatherhead School of Public Health and Tropical Medicine, New Orleans, LA USA

**Keywords:** Social network analysis, Adolescents with HIV, HIV concealment, Emotional social support, Bullying victimization, Verbal and physical abuse

## Abstract

Adolescents with HIV (AWH) often conceal HIV due to stigma and fear of rejection. Stress-buffering and social embeddedness theory suggest that HIV concealment can increase victimization due to a decline in emotional social support (ESS). Understanding the moderating role of ESS between HIV concealment and victimization may help inform interventions to reduce victimization among AWH. A cross-sectional survey including egocentric social network data was conducted among 129 AWH (10–19 Years) at a tertiary care centre in Pune, India between September 2021 and December 2023. The association between HIV concealment and victimization was examined by using a Generalized Linear Model for Negative Binomial (GLMNB) and incidence rate ratios (IRR) were calculated. Moderation was assessed by introducing ESS as a two-way interaction term in the subsequent models between HIV concealment and victimization. A one standard deviation increase in proportion of network members from whom HIV was concealed was associated with a 17% decline in exposure to verbal abuse (IRR = 0.83, 95% CI: 0.66–1.03, p-value = 0.09). A one standard deviation increase in ESS was associated with a 36% decline in physical abuse (IRR = 0.64, 95% CI: 0.44–0.92, p-value = 0.02) and a 22% decline in bullying victimization (IRR = 0.78, 95% CI: 0.63–0.96, p-value = 0.02). ESS moderated the relationship between HIV concealment and victimization, such that high ESS was protective for verbal abuse and low ESS was protective for bullying victimization. Findings highlight the importance of strengthening ESS while addressing stigma to reduce victimization among AWH.

## Introduction

A concealable stigmatized identity refers to an identity that can be hidden to avoid social devaluation, which includes chronic illness (e.g., HIV), sexual gender minoritized identity, and mental illness [1]. While concealment is often used as a protective strategy against stigma, it is associated with poor quality of life [1, 2]. Globally, studies indicate that adolescents with HIV (AWH) engage in concealment to navigate pervasive stigma and to prevent victimization [2–4]. However, identity concealment is a risk factor for internalizing and externalizing problems among children and adolescents [5, 6], which has been associated with higher rates of victimization [7, 8]. For example, child externalizing behaviors can predict severe maltreatment as caregivers can perceive these behaviors as needy or disobedient [7, 8].

The social embeddedness and stress-buffering theories provide a framework to explain the relationship between concealment and victimization [9]. According to the social embeddedness theory, social networks offer stable relationships, social support and provide a safety net where network members can intervene to prevent negative experiences such as victimization [9]. For example, according to a study among Taiwanese students, close knit peer networks were associated with lower levels of bullying victimization as they offered increased peer intervention [10]. Moreover, concealment is considered a negative coping style in adolescent development literature [11], which can reduce embeddedness and increase social isolation and loneliness [11, 12]—established risk factors for victimization [13]. For example, concealing stressors from peers has been associated with higher peer victimization over time [14].

According to stress-buffering theory, social support prevents adverse health outcomes or behaviors by reappraisal of stressful events, inhibition of maladaptive responses, or facilitation of adaptive responses to stressors [9]. While no studies have examined the protective role of social support in the concealment-victimization relationship among AWH, studies have examined the buffering role of social support between concealing sexual gender minoritized identity and mental health. For example, peer and family support has been shown to mitigate the effects of daily concealment of sexual gender minority identity on the mental health of adolescents [15].

A challenge for AWH is that HIV concealment can erode social relationships, reduce social support [16], and increase social isolation [17, 18]. This is evident from numerous studies that have established a positive relationship between HIV disclosure and social support. According to a metanalysis of 21 studies, disclosure of HIV was positively associated with social support [19]. Since HIV concealment can reduce access to network members available for intervention during victimization, increase social isolation, and cause behavioral shifts that can increase (e.g., internalizing and externalizing problems) or decrease (e.g., stress-buffering) victimization, an examination of the moderating role of social support in the HIV concealment-victimization relationship can help inform interventions to reduce victimization among AWH.

**Fig. 1:**
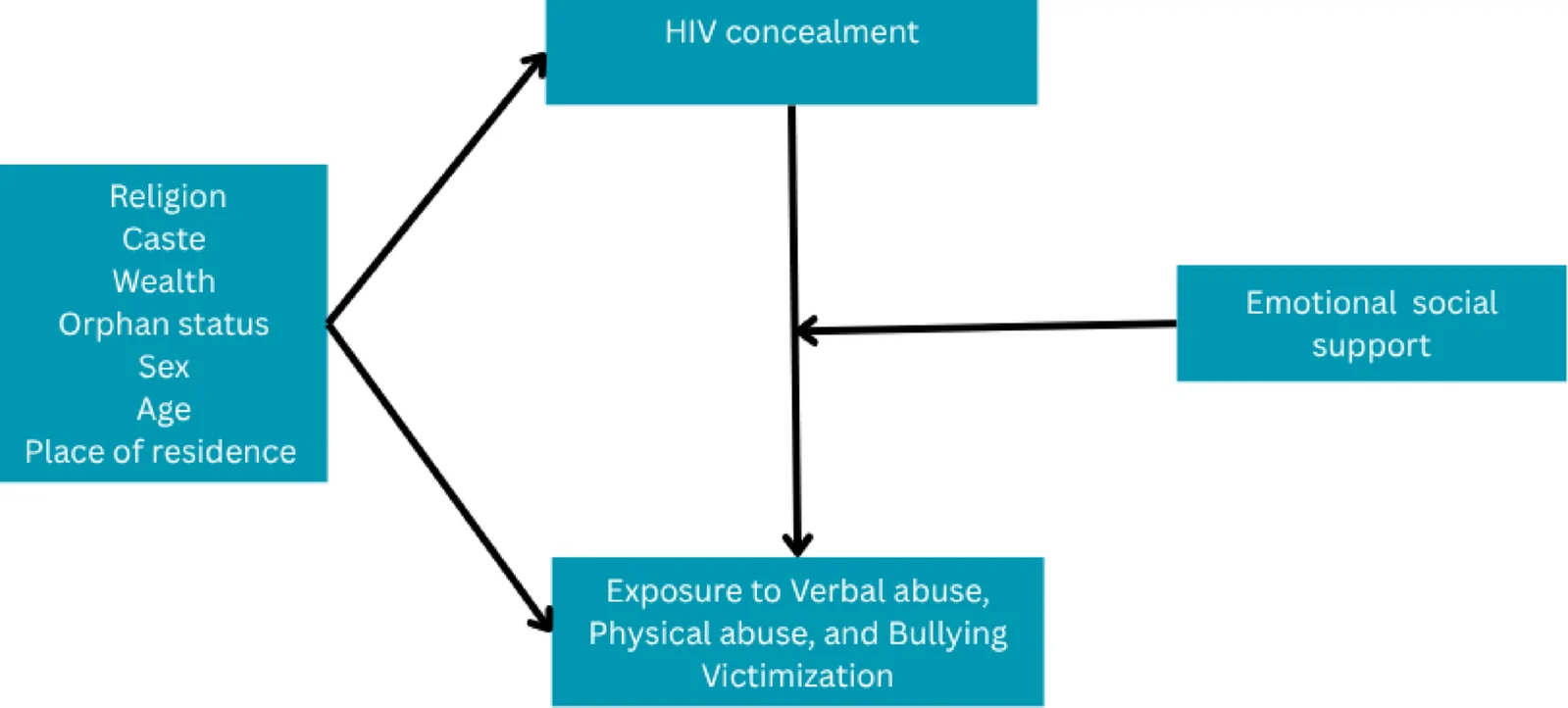
Conceptual model to examine the relationship between HIV concealment, victimization, and emotional social support

We followed an egocentric social network (ESN) approach to understand the buffering role of social support between concealment and victimization among AWH. ESN focuses on attributes of the network rather than the individual level characteristics contributing to the flow of resources and includes both structural (e.g., composition of the network) and functional (e.g., types of support) aspects [20, 21]. Social support is typically divided into subtypes that include emotional, instrumental, appraisal, and informational support [21, 22]. Emotional support relates to love, care, and empathy available from others. Instrumental support is the aid or assistance with tangible needs such as getting to health appointments [21, 22]. Appraisal support relates to help with decision making and providing appropriate feedback. Informational support relates to provision of advice in service of particular needs [21, 22]. To understand the buffering role of social support we focused on emotional social support (ESS), which is a potent buffer against stigma related stressors as indicated by previous research [23]. Given this background, the purpose of this study was to examine the relationship between HIV concealment and victimization and the moderating role of ESS (Fig. 1).

The specific objectives and hypotheses were:

1. To describe the relationship between HIV concealment and exposure to verbal, physical abuse at home and bullying victimization by peers.

We hypothesized that HIV concealment would be positively associated with exposure to verbal, physical abuse at home and bullying victimization by peers.

2. To test the moderating role of ESS in the relationship between HIV concealment and exposure to verbal, physical abuse at home and bullying victimization by peers.

We hypothesized that the association between HIV concealment and verbal, physical abuse and bullying victimization will be weaker for AWH with a high level of ESS compared to those with a low level of ESS.

## Methods

A cross-sectional survey was conducted with 129 AWH at a tertiary care institute and an Antiretroviral Therapy (ART) link center led by people with HIV in Pune, India between September 2021 and December 2023. Only those AWH that were aware of their HIV status were included. Information about AWH HIV awareness was obtained from healthcare providers and outreach workers at the ART link center. At the time of enrollment, a parent or caregiver of AWH below 18 years of age provided consent. Assent was obtained from AWH 10–17 years of age. Assent from AWH and consent from a parent were obtained separately to avoid parental influence on AWH decision to participate in the study. AWH were approached only after parental consent. Ethical approvals for the study were obtained from the tertiary care institute in India, Tulane University and Johns Hopkins University.

**Fig. 2:**
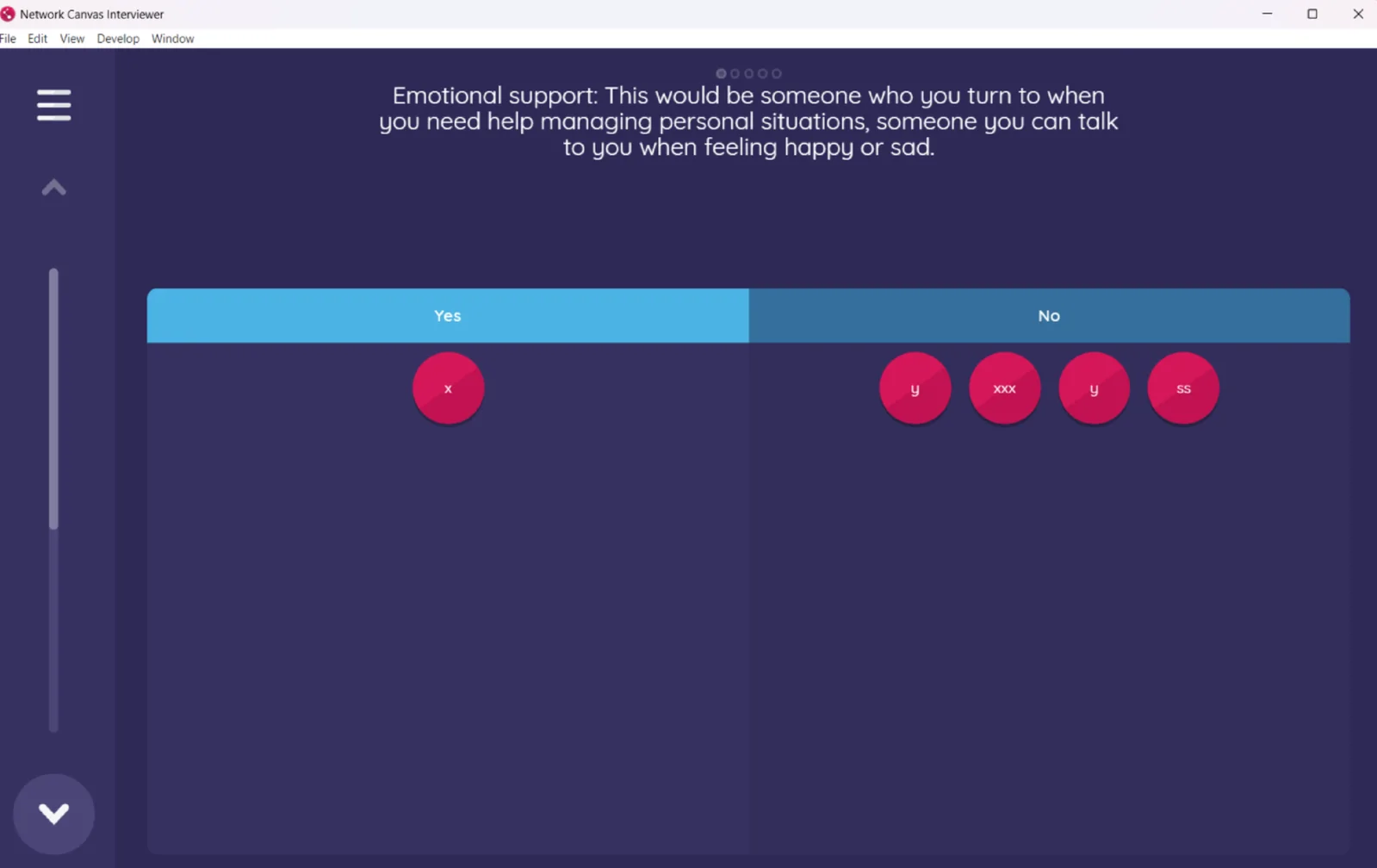
Network canvas interface

 Two trained social scientists collected the survey data through a face-to-face interview. The survey data was recorded in RedCap™ on tablets [24], and the network interviews were conducted through Network Canvas [25]. Network Canvas is a user-friendly platform that allows social network data to be collected interactively, reducing participant burden. Network Canvas uses a point and click graphical user interface that simplifies the data collection [25]. In Network Canvas the protocol is designed in stages, and most stages focus on a single variable type (e.g., ordinal or categorical) to reduce participant burden [25]. For instance, we used categorical variables to categorize ESS (Yes and No) and participants were able to tap the screen to sort network members into categories based on whether or not they offered ESS (Fig. 2). It also includes features such as use of sociogram to visualize network members that participants can connect based on the presence of ties between them. These interactive features significantly cut down the time required to gather network data.

### Study Measures

#### Study Outcomes

Victimization was operationalized as verbal abuse, physical abuse, and bullying victimization. The revised conflict tactic scale (CTS) was utilized to measure and score verbal and physical abuse [26]. Participants provided information about the frequency of verbal and physical abuse at home on five and four items, respectively, on a Likert scale from "1" to "8". Participants responded "1" if they experienced violence once in the past year, "2" for twice, "3" if they experienced 3–5 times, "4" for 6–10 times, "5" for 11–20 times, "6" for more than 20 times, "7" if it did not happen last year but happened before, and "8" if it never happened at all. CTS was coded using midpoints and CTS recommended scoring. The response categories are converted into numerical values or midpoints representing the number of times the act occurred. The midpoints for response options "8" and "7" were 0 and 1, respectively. The midpoint was the same as the response options "1" and "2". For response option "3," the midpoint was 4; for response option "4," it was 8; for "5," it was 15, and for "6" it was 25. Summative scores were created for verbal and physical abuse by adding midpoints.

The modified Olweus bullying scale was utilized to measure bullying victimization in the past 6 months [27]. Eleven items were used to measure the bullying experiences on a Likert scale of "1" to "6". Where the response option "1" was once or twice, "2" was two or three times, "3" was about once a week, "4" was several times a week, "5" was not in the past few months but before, and "6" if it never happened before. Response options "6" were recoded as 0, "5" as 1, "1" as 2, "2" as 3, and "4" as 5. A summative score was created for the recoded response options.

#### Study Exposures

ESN data were collected to measure network characteristics including proportion of network members from whom HIV was concealed and proportion of network members providing ESS. During the social network interview, each participant or ego enumerated up to 10 alters, resulting in 1015 alters in the sample. The number of alters was limited to 10, which is enough to accurately reflect the network measures found in larger samples [22]. Participants named the network members that provided ESS and those that were unaware of their HIV status. After participants first listed all the people in their social network (alters), they were then asked to identify which of these individuals possessed specific characteristics relevant to the study. For example, participants indicated which alters provided ESS by categorizing each network member as either offering or not offering such support. Participants selected alters that provided ESS by helping them manage their emotions and alters they could turn to when sad or upset. This step allowed for the sorting and classification of alters based on attributes such as ESS, which was facilitated using the Network Canvas platform’s interactive interface.

ESS was operationalized as the proportion of network members providing ESS and created by dividing the number of alters providing it by the total network members. Participants also selected alters that were not aware of their HIV status. HIV concealment was operationalized as the proportion of network members from whom HIV was concealed and created by dividing the number of alters concealed by total network members.

#### Covariates

Socio-demographic variables of age, sex, orphan status, place of residence, caste, religion, and household assets were examined as confounders given their potential relation to both exposure and outcomes of interest. Age was measured as a continuous variable of years in age and was dichotomized to below 17 years and 17 years or above. Sex was operationalized as biological sex and measured as a categorical variable coded as male and female. Orphan status was defined as loss of both parents [28] and was measured as a dichotomous variable of both parents deceased or both alive. All orphaned AWH included in the study were in the care of an adult caregiver. Caste was measured in five categories- general, other backward classes, scheduled castes, scheduled tribes, and others- utilized in the national level surveys in India [29].

The government of India has adopted these caste categorizations for affirmative action for greater inclusion of historically marginalized sections of society [30], and we employed as such in the survey so that participants would recognize groupings. Scheduled caste, scheduled tribe, and Others were collapsed into one category, creating three caste categories. Religion was measured in four categories- Hindi, Muslim, Christian, and Others. Religion was dichotomized into majority and minoritized, with Muslim, Christian, and Others categorized as a minoritized religion, similar to previous research in the context [29]. The assets were determined by creating a summative score based on ownership of 14 essential commodities based on previous studies [31, 32] and dichotomized by a cut-off determined by the median value into high and low.

### Analytical Strategy

Descriptive, bivariable, and multivariable analyses were conducted. Mean and standard deviation were calculated for the continuous variables and percentages for the categorical variables. Bivariate analyses examined the crude association between the key variables and the study outcomes. Spearman correlation was implemented to assess the strength and direction of these associations. Correlations were tested at p-value < 0.05 and p-value < 0.10. The multivariable analysis examined the adjusted associations between the methodological variables, and outcomes were examined using a Generalized Linear Model for Negative Binomial (GLMNB). Incidence rate ratios (IRR) were utilized to assess the association, and the null hypothesis was tested at a confidence interval of 95%. Associations were also tested at p-value < 0.19 to ensure that potentially important variables are not prematurely excluded from the model [33]. The *Mass* package in R was utilized to implement the GLMNB.

Moderation was assessed by introducing ESS as a two-way interaction term in the subsequent models between HIV concealment and victimization. Significant interaction terms at p-value < 0.20 following the Aiken and West criteria indicated moderation [34]. *Emtread* command was utilized to investigate the slope of the relationship between concealment and victimization in the quantiles of ESS. These quantiles were − 0.21, −0.08, and 0.21. The moderation was visualized by utilizing the *ggplot2* package.

To address potential multicollinearity and ensure the interpretability of main effects in the presence of interaction terms, all continuous network measures (proportion of network members from whom HIV was concealed and proportion of network members providing ESS) were standardized prior to analysis. This approach follows the Aiken and West criteria for moderation analysis, where standardization reduces structural multicollinearity between primary exposure variables and their interaction products [34].

## Results

Sample characteristics are presented in Table 1. Data were missing on caste, religion, verbal abuse, physical abuse, and bullying victimization, making the analytical total 123. Most of the participants were male (59.35%), resided in urban areas (63.40%), reported low household assets (64.00%), belonged to minoritized castes (48.00%), and majority religion (74.80%). On average, AWH concealed HIV from over half of their network members (M = 0.52, SD = 0.26), and ESS was provided by less than half of the network members (M = 0.46, SD = 0.27). The mean scores of verbal abuse, physical abuse, and bullying victimization were 21.85 (SD = 27.16), 1.79 (SD = 3.86), and 5.55 (SD = 6.31), respectively.

**Table 1:** Socio-demographic characteristics of the study population (*N* = 123)

			% (*N*)	
Age				
Below 17 Years			45.05% (56)	
17 Years or above			54.50% (67)	
Sex				
Female			40.65% (50)	
Male			59.35% (73)	
Place of residence				
Urban			63.40% (78)	
Rural			36.60% (45)	
Orphan status				
Yes			38.20% (47)	
No			61.80% (76)	
Caste†				
General			29.30% (36)	
Other backward classes			22.80% (28)	
Scheduled tribe/class/Others			48.00% (59)	
Religion				
Majority			74.80% (92)	
Minoritized			25.20% (31)	
Household assets				
High			35.00% (43)	
Low			64.00% (80)	

		Mean (SD)		
Network members from whom HIV was concealed (Proportion)		0.52 (0.26)		
Network members providing emotional social support (Proportion)		0.46 (0.27)		
Verbal abuse score		21.85 (27.16)		
Physical abuse score		1.79 (3.86)		
Bullying victimization score		5.55 (6.31)		

†Caste categorization is based on the Government of India’s categorization

The results of the Spearman correlation matrix are detailed in Table 2. Higher concealment was associated with lower verbal abuse (ρ = −0.18, p-value = 0.05) and bullying victimization (ρ = −0.15, p-value = 0.09). ESS was negatively associated with physical abuse (ρ = −0.20, p-value = 0.03) and bullying victimization (ρ = −0.16, p-value = 0.08).

**Table 2:** Correlation between victimization, HIV concealment, and emotional social support (coefficient ρ, (p-value))

	I	II	III	IV	V	VI	VII	VIII	IX	X	XI	XII
						Sex	Place of residence	Orphan status	Caste	Religion	Wealth	Age
Verbal abuse (I)		0.40*	0.29*	−0.18*	−0.11	0.04	0.10	0.07	0.09	0.05	−0.14	0.11
		(0.00)	(0.00)	(0.05)	(0.24)	(0.66)	(0.29)	(0.43)	(0.30)	(0.58)	(0.19)	(0.24)
Physical abuse (II)			0.38**	−0.04	**-**0.20*	0.10	0.12	0.09	0.12	0.11	−0.06	−0.02
			(0.00)	(0.68)	(0.03)	(0.29)	(0.18)	(0.31)	(0.19)	(0.24)	(0.54)	(0.59)
Bullying victimization (III)				−0.15**	−0.16**	−0.13	0.07	0.05	0.25*	−0.11	0.09	0.11
				(0.09)	(0.08)	(0.14)	(0.43)	(0.57)	(0.00)	(0.24)	(0.54)	(0.24)
HIV concealment (IV)					−0.01	0.22*	−0.18*	−0.13	−0.07	0.03	0.05	0.01
					(0.93)	(0.01)	(0.04)	(0.16)	(0.47)	(0.78)	(0.60)	(0.93)
Emotional social support (V)						−0.04	−0.03	0.02	−0.07	−0.08	−0.02	0.07
						(0.70)	(0.72)	(0.84)	(0.41)	(0.35)	(0.83)	(0.45)

*N* = 123, **p-value < 0.10, *p-value < 0.05

### Generalized Linear Model

The results of the GLMNB adjusted for covariates are detailed in Table 3. Religion, orphan status, household assets, and age were not included as covariates as these variables were not significantly associated with outcome or exposure, as indicated in the correlation matrix (Table 2). Since caste was associated with bullying victimization (ρ = 0.25, p-value < 0.001) and sex (ρ = 0.22, p-value = 0.01) and place of residence (ρ = −0.18, p-value = 0.04) were associated with HIV concealment, these were included in the GLMNB model.

**Table 3:** Generalized linear model to examine the relationship between HIV concealment and victimization (adjusted model) (*N* = 123)

	Verbal abuse			Physical abuse				Bullying victimization		
	IRR	95% Confidence interval	*p*-value	IRR	95% Confidence interval		*p*-value	IRR	95% Confidence interval	*p*-value
HIV concealment	0.83	(0.66–1.03)	0.09**	0.80	0.56–1.15	0.23		0.90	0.72–1.11	0.33
Emotional social support	0.98	(0.78–1.22)	0.83	0.64	0.44–0.92	0.02*		0.78	0.63–0.96	0.02*
Sex Female vs. Male	1.55	(0.98–2.46)	0.06**	2.04	0.98–4.24	0.06**		0.68	0.44–1.07	0.09**
Urban vs. Rural	1.17	(0.73–1.87)	0.51	1.29	0.60–2.75	0.51		1.10	0.70–1.72	0.10
Backward class† vs. general class Scheduled caste/ tribe/others vs. general class	1.36	(0.73–2.52)	0.33	1.10	0.41–2.97	0.84		1.24	0.68–2.26	0.48
	0.88	(0.53–1.47)	0.63	1.22	0.53–2.78	0.64		1.90	1.15–3.13	0.01*

†Caste categorization is based on the Government of India’s categorization,

p-value < 0.05*, p-value < 0.10**

Given the small sample size excluding these variables ensures model parsimony and reduced the risk of overfitting [35]. Findings indicate that HIV concealment was negatively associated with exposure to verbal abuse at home. A one standard deviation increase in the proportion of network members from whom HIV was concealed was associated with a 17% decrease in exposure to verbal abuse (IRR = 0.83, 95% CI: 0.66–1.03, p-value = 0.09). HIV concealment was also negatively associated with physical abuse at home and bullying victimization by peers, although the results did not reach statistical significance. However, ESS was found to be negatively and significantly associated with physical abuse at home and bullying victimization. A one standard deviation increase in the proportion of network members providing ESS was associated with a 36% decline in exposure to physical abuse (IRR = 0.64, 95% CI: 0.44–0.92, p-value = 0.02) and a 22% decline in bullying victimization (IRR = 0.78, 95% CI: 0.63–0.96, p-value = 0.02).

### Generalized Linear Model with Moderation

The main effects of the GLMNB with moderation are shown in Table 4. ESS was negatively and significantly associated with physical abuse. A one standard deviation increase in the proportion of network members providing ESS was associated with a 36% decline in physical abuse (IRR = 0.64, 95% CI: 0.44–0.92, p-value = 0.02). ESS was also negatively associated with verbal abuse and bullying victimization; however, the results did not reach statistical significance. HIV concealment was negatively and significantly associated with verbal abuse. A one standard deviation increase in the proportion of network members from whom HIV was concealed was associated with a 24% decline in verbal abuse (IRR = 0.76, 95% CI: 0.61–0.94, p-value = 0.01). HIV concealment was also negatively associated with physical abuse and bullying victimization; however, the results did not reach statistical significance.

**Table 4:** Generalized linear model to examine the moderation of Emotional social support between HIV concealment and victimization (adjusted model) (*N* = 123)

	Verbal abuse			Physical abuse			Bullying victimization		
	IRR	95% Confidence interval	*p*-value	IRR	95% Confidence interval	*p*-value	IRR	95% Confidence interval	*p*-value
HIV concealment	0.76	(0.61–0.94)	0.01*	0.79	(0.55–1.13)	0.28	0.87	(0.70–1.08)	0.20
Emotional social support	0.99	(0.80–1.22)	0.90	0.64	(0.44–0.92)	0.02*	0.79	(0.64–0.98)	0.33
HIV concealment* Emotional social support	0.72	(0.59–0.88)	< 0.001*	1.13	(0.80–1.58)	0.49	1.25	(1.02–1.52)	0.03*
Sex Female vs. Male	1.48	(0.95–2.32)	0.08**	2.11	(1.01–4.38)	0.05*	0.69	(0.44–1.07)	0.10
Urban vs. Rural	1.17	(0.74–1.85)	0.50	1.42	(0.66–3.07)	0.37	1.27	(0.81–1.98)	0.31
Backward class† vs. general class Scheduled caste/ tribe/others vs. general class	1.65	(0.91–3.00.91.00)	0.10	1.15	(0.43–3.11)	0.78	1.25	(0.69–2.27)	0.46
	0.99	(0.60–1.63)	0.97	1.24	(0.54–2.83)	0.62	1.92	(1.18–3.15)	< 0.001*

†Caste categorization is based on the Government of India’s categorization,

p-value < 0.05*, p-value < 0.10**

**Fig. 3:**
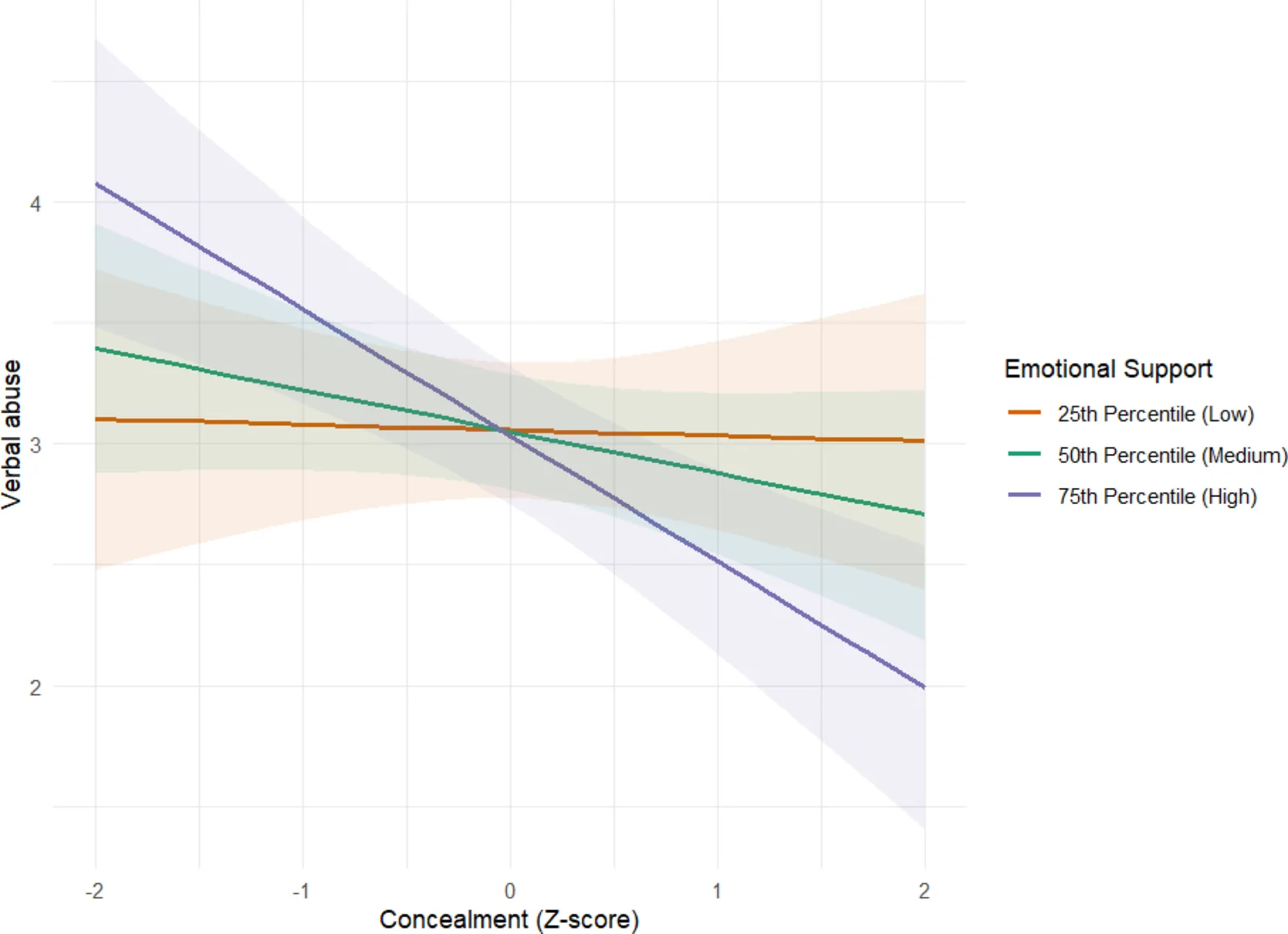
Visual presentation of moderation by emotional social support between HIV concealment and verbal abuse at home

The proportion of network members providing ESS was seen to moderate the relationship between HIV concealment and verbal abuse at home (IRR = 0.72, 95% CI: 0.59–0.88, p-value < 0.01) and bullying victimization by peers (IRR = 1.25, 95% CI: 1.02–1.52, p-value = 0.03). The proportion of network members providing ESS was not seen to moderate the relationship between HIV concealment and physical abuse. As shown in Fig. 3, an increase in proportion of network for whom HIV was concealed was associated with a decline in exposure to verbal abuse for AWH with high, medium, and low ESS (Table 5). AWH with high ESS experienced a sharper decline than those with medium or low ESS, and this result was only statistically significant for the high ESS group (Fig. 3; Table 5).

**Table 5:** Trend analysis of HIV concealment on victimization at different levels of emotional social support (*N* = 123)

Outcome	Level of emotional support	Trend of HIV concealment on outcome	SE	*p*-value
Verbal abuse	25th percentile	−0.02	0.14	0.87
	50th percentile	−0.17	0.12	0.14
	75th percentile	−0.52	0.13	< 0.001*
Bullying victimization	25th percentile	−0.32	0.14	0.02*
	50th percentile	−0.21	0.12	0.06**
	75th percentile	0.03	0.13	0.84

p-value < 0.05*, p-value < 0.10**

**Fig. 4:**
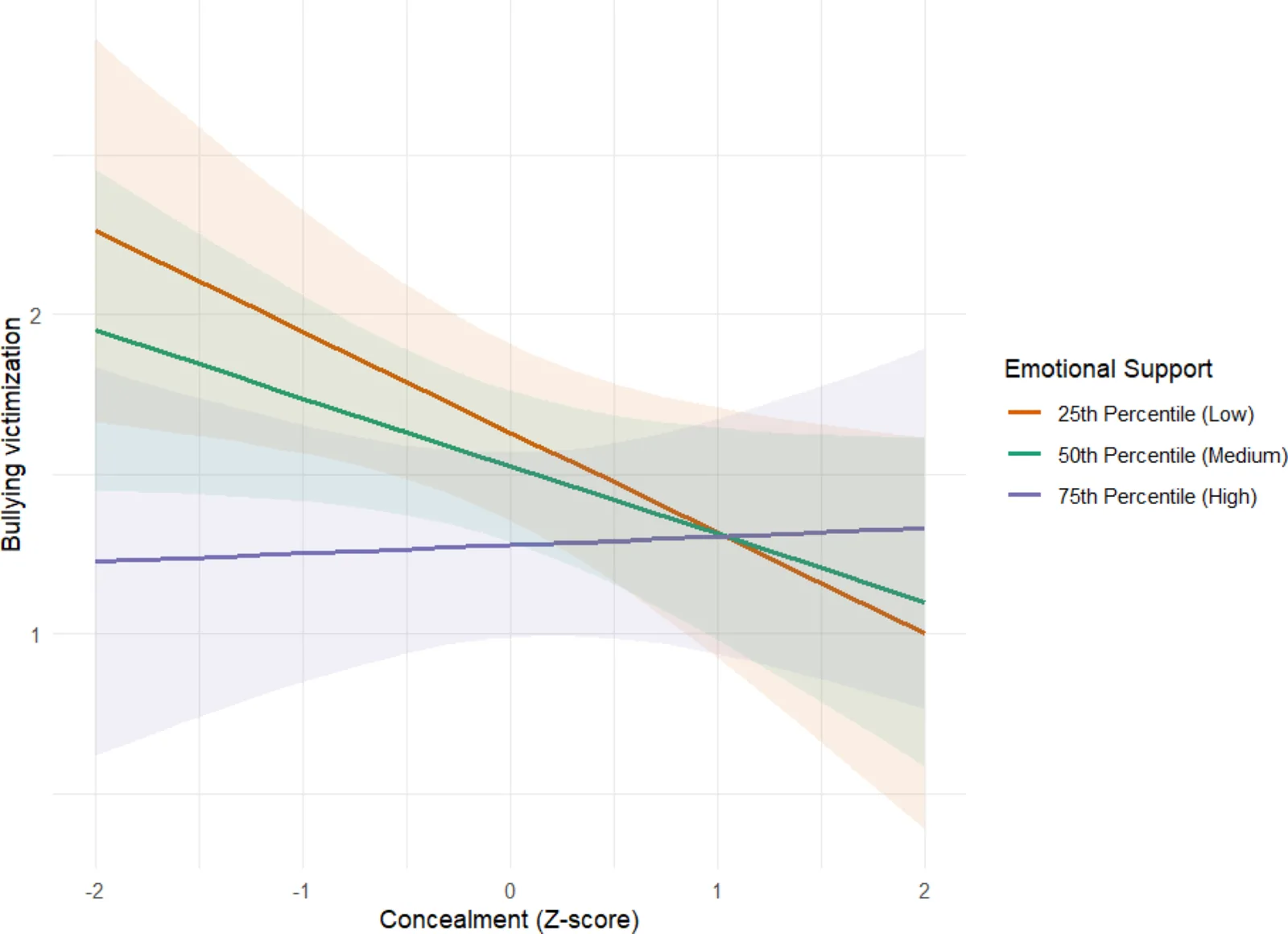
Visual presentation of moderation by emotional social support between HIV concealment and bullying victimization

 As shown in Fig. 4, an increase in the proportion of network members from whom HIV was concealed was significantly associated with a decline in bullying victimization for AWH with medium and low ESS. The decline was lower among AWH with medium ESS. We did not observe a decline in bullying victimization with an increase in concealment among AWH with high ESS.

## Discussion

The objective of this study was to examine the association between HIV concealment and victimization, as well as the moderating role of ESS between HIV concealment and victimization among AWH, the first study to examine the moderating role of ESS. We did not find support for the hypothesis that HIV concealment is associated with an increase in victimization (Hypothesis I). On the contrary, HIV concealment was associated with lower verbal abuse in the home. These findings align with previous research suggesting that HIV concealment can be protective against HIV stigma [36]. We found that AWH had concealed HIV from 52% of their networks. Studies in the US (60%) and Uganda (34%) have reported varying rates of concealment among AWH [37]. Similar to our study, research among gender minoritized groups in the US [38], China [39], and Europe [40], found concealment to be inversely related to victimization. For example, high levels of concealment has been associated with lower levels of victimization in a survey among transgenders in the US [38].

While concealment might be protective against immediate victimization due to HIV stigma, the burden of concealment, including social isolation, contributes to chronic stress that adversely impacts wellbeing [5, 41, 42]. It is key to consider the moderating role of ESS, while interpreting these findings. In our study, the protective effect of concealment on victimization was dependent on the level of ESS available, and ESS moderated the relationship between concealment and both verbal abuse and bullying victimization. Therefore, the implications for wellbeing should be considered within the context of how ESS interacts with concealment to influence outcomes.

Consistent with stress-buffering and social embeddedness theory, we found partial support for our second hypothesis that ESS moderates the relationship between HIV concealment and victimization. Specifically, high ESS was protective for verbal abuse, but low ESS was protective for bullying victimization. The protective role of ESS in verbal abuse suggests that supportive relationships may buffer the psychological strain associated with secrecy. Research suggests that AWH who receive higher levels of ESS have better emotional regulation [43, 44] which might aid in navigating family interactions, and reduce behavioral shifts (e.g., behaviors like slamming doors and emotional outbursts) that could precipitate interpersonal conflict within the household.

High ESS might be protective for verbal abuse due to ESS co-existing with heightened parental monitoring or control and reverse causality between ESS and verbal abuse. One possible mechanism is overprotective parenting where caregivers intensify supervision and verbal discipline to enforce secrecy and prevent disclosure. According to Verma et al., overprotective parenting to prevent HIV disclosure was reported by AWH in India [45]. According to a socialization perspective, such practices may be intended to protect youth from stigma but may contribute to emotional harm [46, 47]. This indicates how ESS and control overlap in family settings, particularly, where HIV stigma remains high. According to reverse causality, rather than ESS being a risk factor for abuse, it may be that adolescents who experience verbal abuse at home may seek ESS from other network members after the exposure to abuse. This is supported by literature suggesting that individuals received greater social support after disclosure of violence [48, 49]. Because this study is cross-sectional, it captures a single point in time and cannot determine which occurred first: the abuse or the increase in ESS.

With respect to bullying victimization, concealment was protective for AWH with low ESS. Concealment might be protective due to the ease of maintaining privacy among smaller, less personal networks, which reduces the risk of passive disclosure [36]. Concealment from less supportive networks might be associated with fewer internalizing problems such as poor self-esteem that are established risk factors of bullying victimization [50, 51]. Limited research suggests that individuals with concealed identities might withdraw from social networks or seek support from other minoritized individuals in the social network [52, 53]. This indicates a reliance on smaller emotional support networks. An examination of the quality of emotional support and the proportion of emotional support provided by HIV-positive network members might improve our understanding of concealment and social support in stress buffering and its impact on victimization.

In our study, ESS was inversely associated with physical abuse and bullying victimization [54]. Findings are supported by other studies suggesting that peer and family support are associated with a decrease in bullying victimization among adolescents in Korea [10] and Africa [55]. Although research has explored social support in victimization, the link between adolescent ESS and physical abuse remains unclear. Studies indicate that low parental ESS corresponds with higher rates of youth physical abuse [41]. Similarly, weak peer networks may reflect this lack of support, potentially contributing to abuse at home.

The protective role of concealment on victimization and the moderating role of ESS indicate the interconnected nature of HIV concealment, ESS, and victimization. Concealment shapes access to social support by limiting disclosure opportunities, while the quality of ESS influences whether concealment is experienced as protective or stressful. At the same time, experiences of victimization may reinforce concealment practices and affect help-seeking behaviors. This suggests a reciprocal relationship between concealment, social support, and victimization, rather than operating as isolated factors. Understanding this interdependence is essential for interpreting the mixed protective and harmful effects of concealment observed in this study.

An improved understanding of the role of ESS is crucial for developing interventions aimed at reducing victimization among AWH. Psychosocial interventions to prevent victimization among AWH should focus on improving family and peer support and management of AWH disclosure concerns. Disclosure counselling should focus on building trust within network members as trust is associated with better disclosure outcomes among social networks [56]. Greater involvement of parents in AWH disclosure counselling is required to reduce enforcement of secrecy by educating parents about the risk of control and overprotective parenting. Interventions should focus on strengthening peer support networks which may reduce both bullying victimization and verbal abuse at home. Community level stigma reduction is also essential as it may reduce the perceived need for concealment and improve social support.

While this study provides important recommendations for psychosocial interventions, there are some limitations. First, the causality and directionality between concealment, ESS, and victimization cannot be determined due to the study’s cross-sectional design. Longitudinal studies are crucial for examining these relationships. Second, we could not account for the differential role of social network characteristics, such as ESS by HIV-positive network members, in the relationship between concealment and victimization. Nor did we account for the confounding role of overprotective parenting. Third, the sample may not be generalizable to all AWH, as the study is limited to perinatally infected AWH retained in care in one state in India.

Fourth, we acknowledge selection bias due to the purposive design. Selection bias includes the participation of AWH with greater integration in the hospital and clinics which may impact their access to social support, conflating the study findings. The findings might also be conflated due to the exclusion of AWH lost to follow-up, which may also be related to victimization. Finally, the self-reporting nature of the study data collection tools might increase social desirability and recall bias. Future research should address these limitations.

## Conclusions

In conclusion, this is the first study to examine HIV concealment among AWH in the Indian context and its relationship to both victimization and ESS. Findings indicate that while HIV concealment was associated with less verbal abuse at home, the relationship between HIV concealment and victimization was dependent on the availability of ESS, particularly for verbal abuse and bullying victimization. Findings indicate that enhancing ESS networks of AWH might reduce victimization. For instance, disclosure counseling in clinics may benefit from understanding the disclosure needs of AWH and provide support in navigating concealed social networks. It could be beneficial to address parental concerns about AWH disclosure, which might be a risk factor for victimization at home. For an increased impact of social support interventions, sustained efforts to reduce stigma at the community level and disclosure concerns at the individual level are recommended. This study contributes novel evidence on how ESS moderates HIV concealment and victimization among adolescents in India, offering a framework for context-sensitive interventions.

## Data Availability

The data that support the findings of this study are available on request from the corresponding author. The data is not publicly available due to privacy or ethical restrictions.
